# Using new indices to predict metabolism dysfunction-associated fatty liver disease (MAFLD): analysis of the national health and nutrition examination survey database

**DOI:** 10.1186/s12876-024-03190-2

**Published:** 2024-03-15

**Authors:** Xu Ming Li, Song Lian Liu, Ya Jun He, Jian Chang Shu

**Affiliations:** 1 Department of Gastroenterology, Guangzhou Red Cross Hospital(Guangzhou Red Cross Hospital of Jinan University), Jinan University, Guangzhou, China; 2 Department of Hepatology, Guangzhou Eighth People’s Hospital, Guangzhou Medical University, Guangzhou, China

**Keywords:** Metabolic dysfunction-associated fatty liver, ALB/GGT, AIP, UHR, Predictive models, NHANES

## Abstract

**Background:**

Metabolism dysfunction-associated fatty liver disease (MAFLD), is the most common chronic liver disease. Few MAFLD predictions are simple and accurate. We examined the predictive performance of the albumin-to-glutamyl transpeptidase ratio (AGTR), plasma atherogenicity index (AIP), and serum uric acid to high-density lipoprotein cholesterol ratio (UHR) for MAFLD to design practical, inexpensive, and reliable models.

**Methods:**

The National Health and Nutrition Examination Survey (NHANES) 2007–2016 cycle dataset, which contained 12,654 participants, was filtered and randomly separated into internal validation and training sets. This study examined the relationships of the AGTR and AIP with MAFLD using binary multifactor logistic regression. We then created a MAFLD predictive model using the training dataset and validated the predictive model performance with the 2017–2018 NHANES and internal datasets.

**Results:**

In the total population, the predictive ability (AUC) of the AIP, AGTR, UHR, and the combination of all three for MAFLD showed in the following order: 0.749, 0.773, 0.728 and 0.824. Further subgroup analysis showed that the AGTR (AUC1 = 0.796; AUC2 = 0.690) and the combination of the three measures (AUC1 = 0.863; AUC2 = 0.766) better predicted MAFLD in nondiabetic patients. Joint prediction outperformed the individual measures in predicting MAFLD in the subgroups. Additionally, the model better predicted female MAFLD. Adding waist circumference and or BMI to this model improves predictive performance.

**Conclusion:**

Our study showed that the AGTR, AIP, and UHR had strong MAFLD predictive value, and their combination can increase MAFLD predictive performance. They also performed better in females.

## Background

Metabolism dysfunction-associated fatty liver disease (MAFLD) is hepatic steatosis with overweight/obesity, type-2 diabetes, or metabolic dysregulation, as described at an international expert consensus meeting [1–3]. MAFLD is the most common chronic liver disease, which raises the risk of cardiovascular disease [4] and all-cause death [5, 6]. However, as is known, the gold standard of diagnosis for fatty liver is pathology biopsy, which is an invasive procedure with potential negative effects [7]. Thus, it is of high clinical importance and value to investigate practical, straightforward, and reliable predictors of fatty liver disease.

The serum uric acid to high-density lipoprotein cholesterol ratio (UHR) is a recently proposed inflammatory marker that has been shown to be associated with the development of NAFLD, metabolic syndrome, diabetes mellitus, insulin resistance, and cardiovascular risk [8–10]. The plasma atherogenicity index (AIP), defined as the logarithm of the triglyceride to high-density lipoprotein cholesterol (HDL) ratio (TG/HDL-C), is significantly elevated in patients with fatty liver disease and may be a potential indicator for identifying fatty liver disease [11]. It has been previously shown to be associated with NAFLD [12], MAFLD [13, 14], cardiovascular risk [15], and metabolic risk [16]. Albumin (ALB)/alkaline phosphatase (ALP), a biological measure of liver function, has been shown in prior research to be a reliable independent predictor of NAFLD and MAFLD [17]. Intrahepatic cholestasis may be involved in the development of NAFLD or MAFLD [18]. In contrast, sludgy hepatitis can be reflected by direct bilirubin, glutamyl transpeptidase (GGT) and ALP. Albumin transports bilirubin and cholesterol are an important indicators of liver function. Currently, an important and practical indicator used to assess liver function in liver cancer is the ALBI (albumin-bilirubin) score [19], which can be used to predict cirrhosis in the loss-of-compensation phase [20].

Although NAFLD and MAFLD share commonalities, the diagnostic criteria are significantly different, and thus, many predictors of NAFLD still need to be further explored in MAFLD. Consequently, we examined the predictive ability of the AGTR, UHR, AIP and their combination for MAFLD using the National Health and Nutrition Examination Survey (NHANES) 2007–2018 dataset, to design a practical, inexpensive, and reliable predictive tool for MAFLD.

## Methods

### Database

Data were obtained from the NHANES database, which uses a complex, hierarchical, multistage, probabilistic clustering design to assess health and nutritional status in the U.S. All participants provided written informed consent.

### Definitions and inclusion criteria

**Fig. 1:**
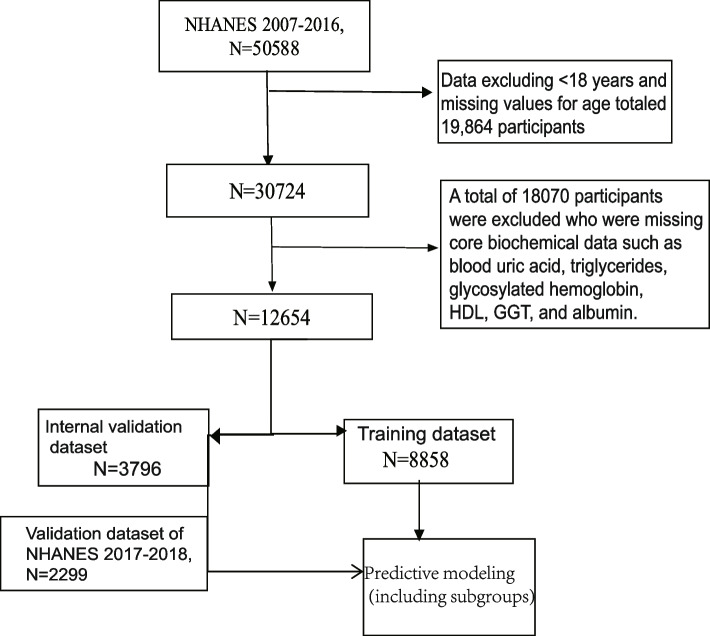
Flow chart

The analysis included subjects 18 years of age or older and included demographics (age, sex, race, poverty-to-income ratio), triglycerides, HDL, blood uric acid, GGT, albumin, data relevant to the diagnosis of MAFLD, and a validated FibroScan. After excluding participants with no key biochemistry data (blood uric acid, triglycerides, glycosylated hemoglobin (HbA1c), HDL, GGT, albumin), incomplete transient elastography data and data that were not diagnostic of MAFLD, a total of 12,654individuals were finally enrolled. The NHANES database 2007–2016 cycle dataset ultimately included 12,654 subjects for statistical analyses and predictive modeling, and predictive model validation was performed using the 2017–2018 cycle dataset, with inclusion criteria and a participant stratification algorithm, as shown in Fig. 1.

### Definition of the ending variable MAFLD

The diagnosis of MAFLD is based on histologic (biopsy) imaging or blood biomarker evidence of hepatic fat accumulation (hepatic steatosis) and one of the following three criteria: overweight/obesity, the presence of type-2 diabetes mellitus (T2DM), or evidence of metabolic derangement. Patients with fatty liver are identified by United States Fatty Liver Index (USFLI) ≥30 in the NHANES 2007–2016 dataset or by controlled attenuation parameter (CAP) > 274 [21] in the 2017–2018 dataset and have two of the following items [1]: (i) waist circumference (WC) > 102 cm for men or > 88 cm for women; (ii) blood pressure 130/85 mmHg or related medication; (iii) fasting plasma triglycerides > 1.70 mmol/L or related medication; (iv) plasma high-density lipoprotein (HDL) cholesterol < 1.0 mmol/L for males or < 1.3 mmol/L for females or related medication; (v) preexisting diabetes mellitus (fasting blood glucose 5.6–6.9 mmol/L or hemoglobin A1C 39–47 mmol/mol); and (vi) homeostasis model assessment of insulin resistance (HOMA-IR) score) ≥2.5.

### Definition of research variables

AIP = log10(TG/HDL-c) [22]; UHR = serum uric acid (mg/dL)/HDL-c(mg/dL);AGTR = albumin (g/L)/GGT (U/L).

### Other variable definitions

Participants with at least one of the following conditions are defined as having diabetes in the NHANES: 1. diagnosed with diabetes by a self-reported prior physician or currently being treated for glycemic control (use of insulin or oral hypoglycemic agents); and 2. laboratory results met the following criteria: 1) glycated hemoglobin ≥ 6.5% and 2) fasting blood glucose > 7.0 mmol/L. Hypertension was defined as self-reported physician-diagnosed hypertension or being on prescribed medication. Blood pressure was assessed using an average of 3 consecutive standardized blood pressure readings. Alcohol consumption was categorized as moderate, excessive and no alcohol consumption [23]. Physical activity was defined as follows: 1. Light activity; 2. moderate activity; and 3. High-intensity activity [24]. A “smoker” was defined as an adult who had smoked 100 cigarettes in his or her lifetime, and a “never smoker” was defined as any adult who had never smoked or had smoked fewer than 100 cigarettes in his or her lifetime. Homeostasis model assessment of insulin resistance (HOMA-IR) score = (fasting insulin in mIU/mL) × (fasting glucose in mg/dL)/405 [25]. Total protein intake and vitamin C were extracted from 2-day dietary interview data and averaged over two days. NCHS Ethics Review Board supported the research. Furthermore, written informed consent was received from each subject [26].

### Statistical analysis

The study is consistent with the transparent reporting of a multivariable prediction model for individual prognosis or diagnosis (TRIPOD): the TRIPOD statement [27]. NHANES uses a complex survey design to ensure national representativeness, and data analysis of the complex sampling design was conducted under the guidance of the NCHS. Normally distributed variables are expressed as means (standard deviations) and non-normally distributed variables are expressed as medians (quartiles). Categorical variables are shown as unweighted counts (weighted %). Categorical variables were tested with weighted chi-square tests, continuous variables were tested using t tests for normally distributed data, and nonnormally distributed data were tested using Wilcoxon rank sum tests. Weighted univariate and multivariate logistic regression models were used to identify associations between the study variables and the outcome variable (MAFLD), and the data were displayed as odds ratios (ORs) and 95% confidence intervals (CIs) for unadjusted, partially adjusted, and fully adjusted confounders. The confounders for the partial adjustment are: age, gender, race, and income-poverty ratio, and the full adjustment adds the following factors to the partial adjustment: BMI, physical activity, diabetes mellitus, alkaline phosphatase, mercury, cadmium, transaminases, smoking, drinking, protein intake, vitamin C and LDL. We considered two-sided P values less than 0.05 as indicative of statistical significance. For model development, the NHANES database 2007–2016 cycle dataset (12,654 participants in total) was randomly divided into two groups (8,858 for the training dataset and 3,796 for the internal validation dataset) in a 7:3 ratio. The training dataset was used to develop the model, internal validation was performed using the validation dataset, and secondary validation was performed using the dataset from the NHANES 2017–2018 cycle. The R programming language was used for all data extraction and statistical analyses (R version 4.1.2). The strategy for dealing with missing values of covariates in this study: if the number of missing < 20% used multiple interpolation (mi packages for interpolation), more than 20% were excluded from the data. Missing values for study variables as well as key variables for the diagnosis of MAFLD were simply excluded. Use the gtsummary package to construct the output of the prediction model. The qROC package plots the ROC curve along with the output AUC value.

## Results

### Baseline Characteristics of the Subjects

The baseline characteristics of the subjects are shown in Table 1 to Table 4. In the Tables 1 and 2, of the 12,654 subjects, 4020 patients were diagnosed with MAFLD (32%), with a median age of 53 years, and 8634 were non-MAFLD patients (68%), with a median age of 43 years. ALT, TC, ALP, CRE, BMI, WC, AIP, and UHR were higher in MAFLD patients than in non-MAFLD patients (*p* < 0.05). Tables 3 and 4 show that the training set's baseline information is similar to the internal and secondary validation sets.

**Table 1:** Basic characteristics of participants according to MAFLD from NHANES 2007–2016

Characteristic	Non-MAFLD, *N* = 8634(68%)^1^	MAFLD, *N* = 4020 (32%)^1^	*p*-value^2^
Sex			< 0.001
Male	3,981(45%)	2,231(57%)	
FeMale	4,653(55%)	1,789(43%)	
Age	43 (30, 57)	53 (40, 64)	< 0.001
BMI (kg/m2)	26 (23, 29)	33 (30, 38)	< 0.001
Ratio of income to poverty	2.92 (1.39, 4.94)	2.72 (1.43, 4.67)	0.088
Race			< 0.001
Mexican American	1,421(9.1%)	610(7.9%)	
Other Hispanic	892(5.7%)	547(6.5%)	
Non-Hispanic White	3,465(66%)	1,851(71%)	
Non-Hispanic Black	1,789(11%)	694(8.6%)	
Other Race -	1,067(8.3%)	318(5.7%)	
AIP	-0.15 (0.30)	0.16 (0.31)	< 0.001
AGTR	2.65 (1.95, 3.50)	1.50 (0.96, 2.11)	< 0.001
UHR	0.09 (0.07, 0.12)	0.14 (0.10, 0.17)	< 0.001
TC (mmol/l)	1.01 (0.71, 1.41)	1.65 (1.15, 2.33)	< 0.001
CRE(umol/L)	307 (75)	366 (85)	< 0.001
WC (cm)	92 (12)	114 (14)	< 0.001
Hypertension, n (%)			< 0.001
NO	6,359(77%)	1,917(49%)	
Yes	2,275(23%)	2,103(51%)	
Overweight/Obesity			< 0.001
NO	3,738(44%)	173(3.8%)	
Yes	4,896(56%)	3,847(96%)	
Activity			< 0.001
Light	1,875(18%)	1,270(28%)	
Moderate	3,330(38%)	1,656(42%)	
Vigorous	3,429(44%)	1,094(30%)	
ALB(g/L)	43.1 (3.3)	42.1 (3.2)	< 0.001
Drinking			< 0.001
Never	2,178(20%)	1,147(24%)	
Moderate	745(9.1%)	475(12%)	
Excessive	5,711(71%)	2,398(64%)	
ALP(U/L)	61 (50, 74)	68 (56, 83)	< 0.001
ALT(U/L)	19 (15, 25)	27 (20, 37)	< 0.001
Diabetes, n (%)			< 0.001
NO	8,023(95%)	3,099(81%)	
Yes	611(4.8%)	921(19%)	
Smoking			< 0.001
NO	5,044(58%)	2,050(50%)	
Yes	3,590(42%)	1,970(50%)	
Protein (gm)	82 (34)	84 (35)	0.022
Vitamin C (mg)	68 (33, 117)	57 (27, 107)	< 0.001
Mercury, total (umol/L)	8 (12)	7 (10)	< 0.001
Blood cadmium (nmol/L)	4.5 (5.4)	4.3 (5.3)	0.2

*AGTR* Albumin to glutamyl transpeptidase ratio, *AIP* Plasma atherogenicity index, *UHR* Serum uric acid to high-density lipoprotein cholesterol ratio

^1^n (unweighted)(%); Median (IQR)

^2^chi-squared test with Rao & Scott's second-order correction; Wilcoxon rank-sum test for complex survey samples; t-test adapted to complex survey samples

**Table 2:** Basic characteristics of participants according to MAFLD from NHANES 2017–2018

Characteristic	Non-MAFLD, *N* = 1318(58%)^*1*^	MAFLD, *N* = 981(42%)^*1*^	*p*-value^*2*^
Sex			< 0.001
Male	577(42%)	566(60%)	
FeMale	741(58%)	415(40%)	
Age	42 (28, 58)	53 (38, 64)	< 0.001
BMI (kg/m2)	26 (23, 30)	32 (29, 36)	< 0.001
Ratio of income to poverty	3.04 (1.48, 4.98)	3.41 (1.70, 5.00)	0.039
Race			< 0.001
Mexican American	139(7.0%)	184(12%)	
Other Hispanic	114(6.3%)	102(7.1%)	
Non-Hispanic White	456(64%)	342(62%)	
Non-Hispanic Black	334(12%)	170(7.7%)	
Other Race	275(10%)	183(10%)	
AIP	-0.12 (0.27)	0.18 (0.31)	< 0.001
AGTR	2.59 (1.76, 3.50)	1.65 (1.06, 2.28)	< 0.001
UHR	0.09 (0.07, 0.12)	0.13 (0.10, 0.16)	< 0.001
TC (mmol/l)	1.04 (0.78, 1.52)	1.76 (1.25, 2.45)	< 0.001
CRE(umol/L)	300 (79)	353 (86)	< 0.001
WC (cm)	92 (14)	112 (15)	< 0.001
Hypertension, n(%)			< 0.001
NO	945(78%)	508(56%)	
YES	373(22%)	473(44%)	
Overweight/Obesity			< 0.001
NO	564(43%)	62(4.4%)	
YES	754(57%)	919(96%)	
Activity			< 0.001
Light	278(17%)	267(21%)	
Moderate	408(28%)	361(39%)	
Vigorous	632(56%)	353(39%)	
ALB(g/L)	41.3 (3.3)	40.9 (3.2)	0.2
Drinking			0.5
Never	469(41%)	313(39%)	
Moderate	477(39%)	375(39%)	
Excessive	372(19%)	293(23%)	
ALP(U/L)	69 (56, 85)	77 (64, 94)	< 0.001
ALT(U/L)	16 (12, 22)	22 (16, 32)	< 0.001
Diabetes, n (%)			< 0.001
NO	1,214(96%)	767(82%)	
YES	104(4.2%)	214(18%)	
Smoking			0.6
NO	796(58%)	557(56%)	
YES	522(42%)	424(44%)	
Protein (gm)	80 (38)	84 (37)	0.059
Vitamin C (mg)	62 (28, 107)	57 (26, 104)	0.6
Blood mercury, total (nmol/L)	7 (11)	7 (11)	0.8
Blood cadmium (nmol/L)	4.2 (5.3)	3.5 (4.9)	0.068

*AGTR* Albumin to glutamyl transpeptidase ratio, *AIP* Plasma atherogenicity index, *UHR* Serum uric acid to high-density lipoprotein cholesterol ratio

^1^n (unweighted)(%); Median (IQR)

^2^chi-squared test with Rao & Scott's second-order correction; Wilcoxon rank-sum test for complex survey samples; t-test adapted to complex survey samples

**Table 3:** Basic characteristics of participants according to train and internal validation set

Characteristic	Train Set, *N* = 8858(69%)^*1*^	Validation1, *N* = 3796(31%)^*1*^	*p*-value^*2*^
Sex			0.7
Male	4,354(49%)	1,858(49%)	
FeMale	4,504(51%)	1,938(51%)	
Age	46 (32, 60)	46 (32, 60)	0.8
BMI (kg/m2)	28 (24, 32)	28 (24, 32)	0.8
Ratio of income to poverty	2.81 (1.37, 4.81)	2.96 (1.47, 4.97)	0.10
Race			0.5
Mexican American	1,441(8.9%)	590(8.5%)	
Other Hispanic	1,014(6.0%)	425(5.8%)	
Non-Hispanic White	3,704(67%)	1,612(68%)	
Non-Hispanic Black	1,734(10%)	749(10%)	
Other Race	965(7.6%)	420(7.1%)	
AIP	-0.05 (0.34)	-0.05 (0.33)	0.7
MAFLD			0.3
NO	6,044(68%)	2,590(69%)	
YES	2,814(32%)	1,206(31%)	
AGTR	2.26 (1.48, 3.13)	2.26 (1.52, 3.15)	0.4
UHR	0.10 (0.07, 0.14)	0.11 (0.08, 0.14)	0.5
TC (mmol/l)	1.15 (0.79, 1.72)	1.17 (0.81, 1.75)	0.3
CRE(umol/L)	325 (83)	327 (82)	0.4
WC (cm)	99 (16)	99 (17)	0.9
Hypertension, n (%)			0.3
NO	5,798(68%)	2,478(69%)	
YES	3,060(32%)	1,318(31%)	
Overweight/Obesity			0.8
NO	2,738(31%)	1,173(31%)	
YES	6,120(69%)	2,623(69%)	
Activity			0.6
Light	2,222(21%)	923(21%)	
Moderate	3,472(39%)	1,514(39%)	
Vigorous	3,164(39%)	1,359(41%)	
ALB(g/L)			0.5
Mean (SD)	42.8 (3.3)	42.8 (3.3)	
Drinking			0.8
Never	2,314(21%)	1,011(21%)	
Moderate	862(10%)	358(9.7%)	
Excessive	5,682(69%)	2,427(69%)	
ALP(U/L)	63 (52, 77)	63 (52, 76)	0.10
ALT(U/L)	21 (16, 29)	21 (16, 28)	0.6
Diabetes, n (%)			0.8
NO	7,821(91%)	3,301(91%)	
YES	1,037(9.2%)	495(9.4%)	
Smoking			0.059
NO	4,910(55%)	2,184(57%)	
YES	3,948(45%)	1,612(43%)	
Protein (gm)	83 (34)	83 (35)	0.6
Vitamin C (mg)	64 (30, 114)	65 (31, 115)	0.8
Mercury, total (umol/L)	7 (11)	7 (12)	0.8
Blood cadmium (nmol/L)	4.5 (5.4)	4.3 (5.3)	0.2

*AGTR* Albumin to glutamyl transpeptidase ratio, *AIP* Plasma atherogenicity index, *UHR* Serum uric acid to high-density lipoprotein cholesterol ratio

^1^n (unweighted)(%); Median (IQR)

^2^chi-squared test with Rao & Scott's second-order correction; Wilcoxon rank-sum test for complex survey samples; t-test adapted to complex survey samples

**Table 4:** Basic characteristics of participants according to train and second validation set

Characteristic	Train Set, *N* = 8858(40%)^*1*^	Validation2, *N* = 2299(60%)^*1*^	*p*-value^*2*^
Sex			0.6
Male	4,354(49%)	1,143(50%)	
FeMale	4,504(51%)	1,156(50%)	
Age	46 (32, 60)	47 (32, 61)	0.8
BMI (kg/m2)	28 (24, 32)	28 (25, 33)	0.015
Ratio of income to poverty	2.81 (1.37, 4.81)	3.19 (1.58, 5.00)	0.014
Race			0.5
Mexican American	1,441(8.9%)	323(9.3%)	
Other Hispanic	1,014(6.0%)	216(6.7%)	
Non-Hispanic White	3,704(67%)	798(64%)	
Non-Hispanic Black	1,734(10%)	504(10%)	
Other Race	965(7.6%)	458(10%)	
AIP	-0.05 (0.34)	0.01 (0.32)	< 0.001
MAFLD			< 0.001
NO	6,044(68%)	1,318(58%)	
YES	2,814(32%)	981(42%)	
AGTR	2.26 (1.48, 3.13)	2.11 (1.38, 3.00)	< 0.001
UHR	0.10 (0.07, 0.14)	0.10 (0.07, 0.14)	0.9
TC (mmol/l)	1.15 (0.79, 1.72)	1.32 (0.88, 1.94)	< 0.001
CRE(umol/L)	325 (83)	322 (86)	0.3
WC (cm)	99 (16)	101 (17)	0.045
Hypertension, n (%)			0.6
NO	5,798(68%)	1,453(69%)	
YES	3,060(32%)	846(31%)	
Overweight/Obesity			0.028
NO	2,738(31%)	626(27%)	
YES	6,120(69%)	1,673(73%)	
Activity			< 0.001
Light	2,222(21%)	545(19%)	
Moderate	3,472(39%)	769(33%)	
Vigorous	3,164(39%)	985(49%)	
ALB(g/L)			< 0.001
Mean (SD)	42.8 (3.3)	41.1 (3.3)	
Drinking			< 0.001
Never	2,314(21%)	665(21%)	
Moderate	862(10%)	852(39%)	
Excessive	5,682(69%)	782(40%)	
ALP(U/L)	63 (52, 77)	73 (59, 89)	< 0.001
ALT(U/L)	21 (16, 29)	18 (13, 27)	< 0.001
Diabetes, n (%)			0.3
NO	7,821(91%)	1,981(90%)	
YES	1,037(9.2%)	318(10%)	
Smoking			0.14
NO	4,910(55%)	1,353(57%)	
YES	3,948(45%)	946(43%)	
Protein (gm)	83 (34)	82 (37)	0.5
Vitamin C (mg)	64 (30, 114)	59 (27, 105)	0.013
Mercury, total (umol/L)	7 (11)	7 (11)	0.4
Blood cadmium (nmol/L)	4.5 (5.4)	3.9 (5.2)	0.010

*AGTR* Albumin to glutamyl transpeptidase ratio, *AIP* Plasma atherogenicity index, *UHR* Serum uric acid to high-density lipoprotein cholesterol ratio

^1^n (unweighted)(%); Median (IQR)

^2^chi-squared test with Rao & Scott's second-order correction; Wilcoxon rank-sum test for complex survey samples; t-test adapted to complex survey samples

### AGTR, AIP and MAFLD correlation analysis in NHANES 2007–2016

The UHR has been shown to be associated with NAFLD in many studies [28–33]. Thus, only AIP, AGTR and MAFLD were analyzed for correlation. The results of the analysis are shown in Table 5. In the risk association analysis of AIP quartiles with MAFLD, the AIP was significantly associated with MAFLD in models 1–3. In Model 3, the odds ratio (OR) values of the second, third, and fourth quartiles of AIP were 1.81 (95% CI: 1.35–2.44), 3.32 (95% CI:(2.61- 4.21), and 7.27 (95% CI: 5.47–9.65), respectively, which were significantly different (*P* < 0.001) independent risk factors. The results of logistic regression analysis of the relationship between the AGTR and MAFLD showed that the odds ratios (ORs) in Model 1, Model 2, and Model 3 were 0.31 (95% CI: 0.29–0.33), 0.30 (95% CI: 0.28–0.32), and 0.30 (95% CI: 0.27–0.33), respectively, with a p value of less than 0.001, which indicated a statistically significant difference. The AGTR was an independent protective factor for MAFLD.

**Table 5:** Analysis of the correlation between the study variables and MAFLD from NHANES 2007–2016

Characteristic	OR^*1*^	95% CI^*1*^	*p*-value
**Model1**			
**AGTR**	0.31	0.29, 0.33	< 0.001
AIP			
Q1(≤ -0.2824)	—	—	
Q2(-0.2824, -0.0687)	2.66	2.17, 3.26	< 0.001
Q3(-0.0687,0.1634)	6.16	5.08, 7.46	< 0.001
Q4(> 0.1634)	16.7	13.3, 21.0	< 0.001
**Model2**			
**AGTR**	0.30	0.28, 0.32	< 0.001
AIP			
Q1(≤ -0.2824)	—	—	
Q2(-0.2824, -0.0687)	2.56	2.11, 3.12	< 0.001
Q3(-0.0687,0.1634)	5.88	4.86, 7.11	< 0.001
Q4(> 0.1634)	16.3	13.0, 20.5	< 0.001
**Model3**			
**AGTR**	0.30	0.27, 0.33	< 0.001
AIP			
Q1(≤ -0.2824)	—	—	
Q2(-0.2824, -0.0687)	1.81	1.35, 2.44	< 0.001
Q3(-0.0687,0.1634)	3.32	2.61, 4.21	< 0.001
Q4(> 0.1634)	7.27	5.47, 9.65	< 0.001

Model1 No variables were adjusted

Model2 Adjusted for age, gender, race, and income poverty ratio

Model3 Adjusted for BMI, physical activity, diabetes mellitus, alkaline phosphatase, mercury, cadmium, transaminases, smoking, drinking, protein intake, vitamin C and potential confounders of LDL on a previous basis based on model2

*AGTR* albumin to glutamyl transpeptidase ratio, *AIP* plasma atherogenicity index

^1^*OR *Odds Ratio*, *CI Confidence Interval

### Predictive results of AGTR, UHR, and AIP on MAFLD

**Fig. 2:**
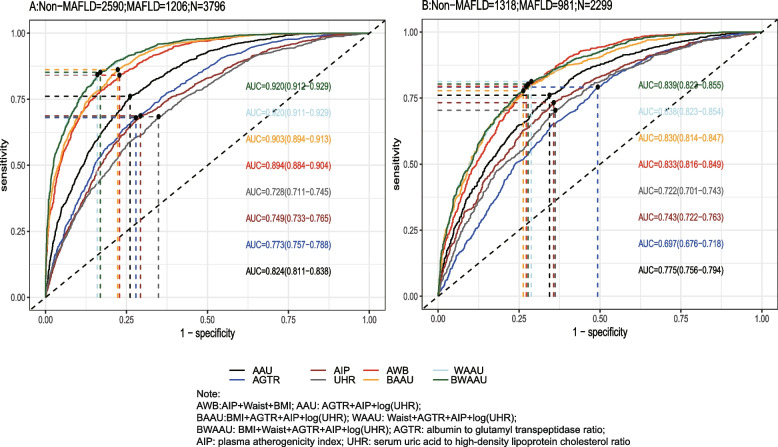
**A**: The roc curve for internal validation; **B**: The roc curve for the second validation

**Fig. 3:**
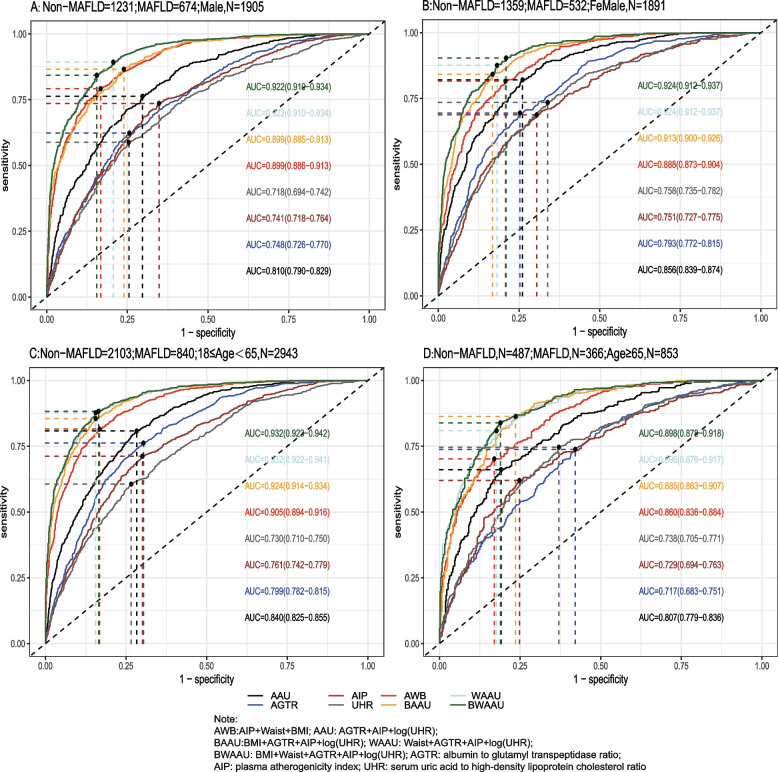
The internal validation roc curve of the subgroup

**Fig. 4:**
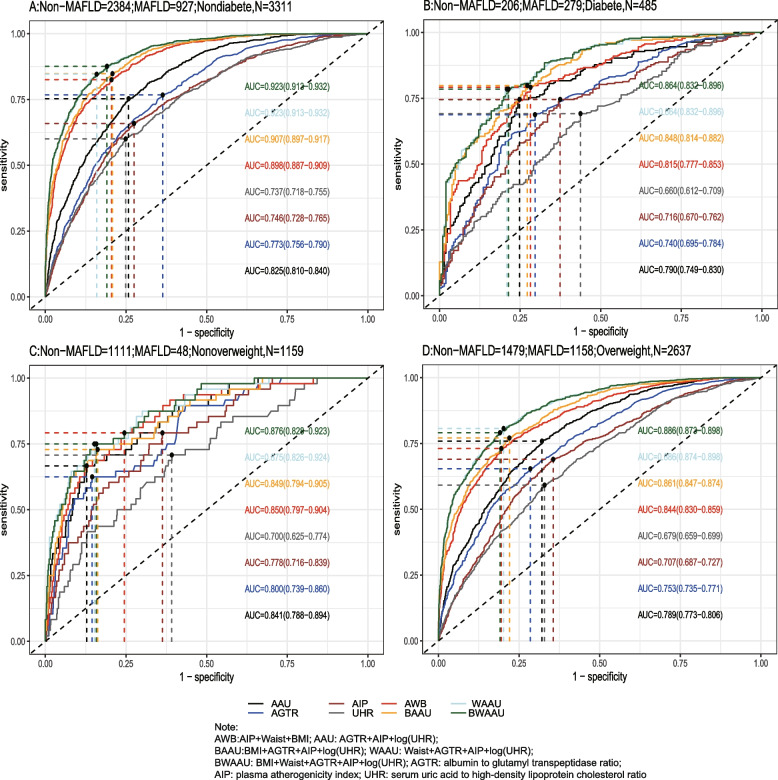
The internal validation roc curve of the subgroup

**Fig. 5:**
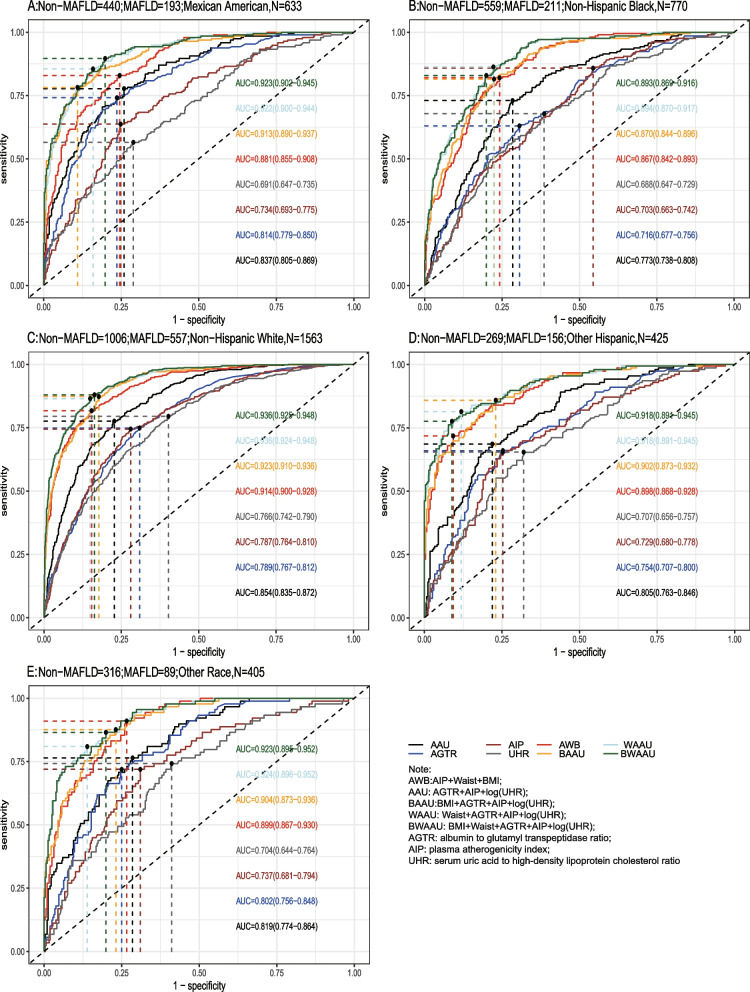
The internal validation roc curve of the subgroup

**Fig. 6:**
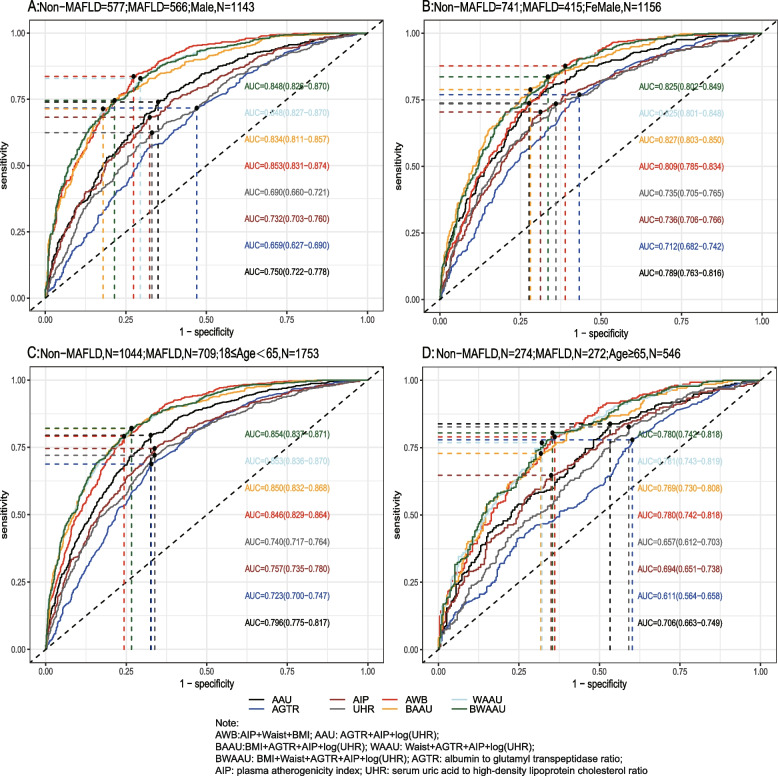
The second validation's roc curve for the subgroup

**Fig. 7:**
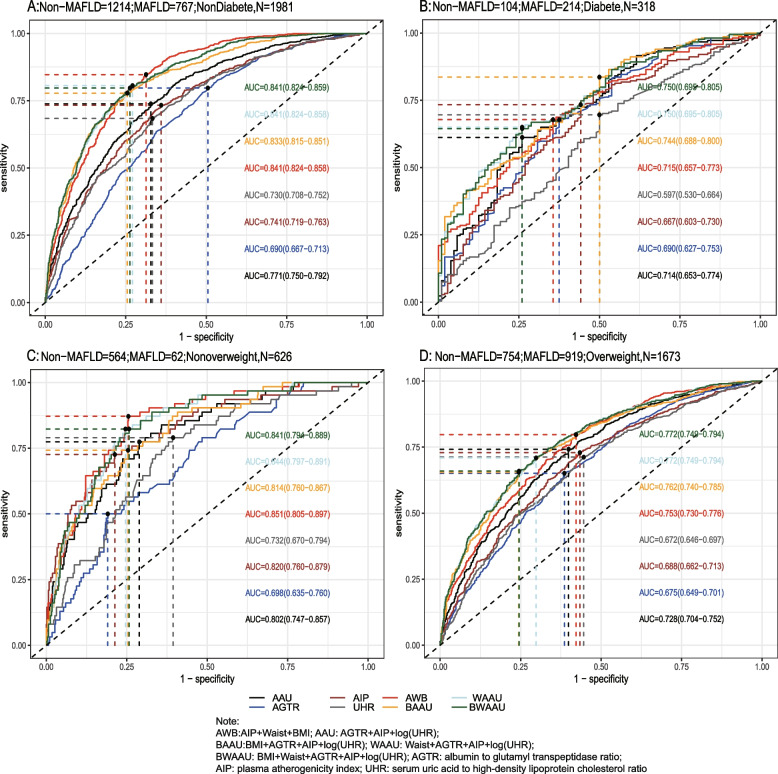
The second validation's roc curve for the subgroup

**Fig. 8:**
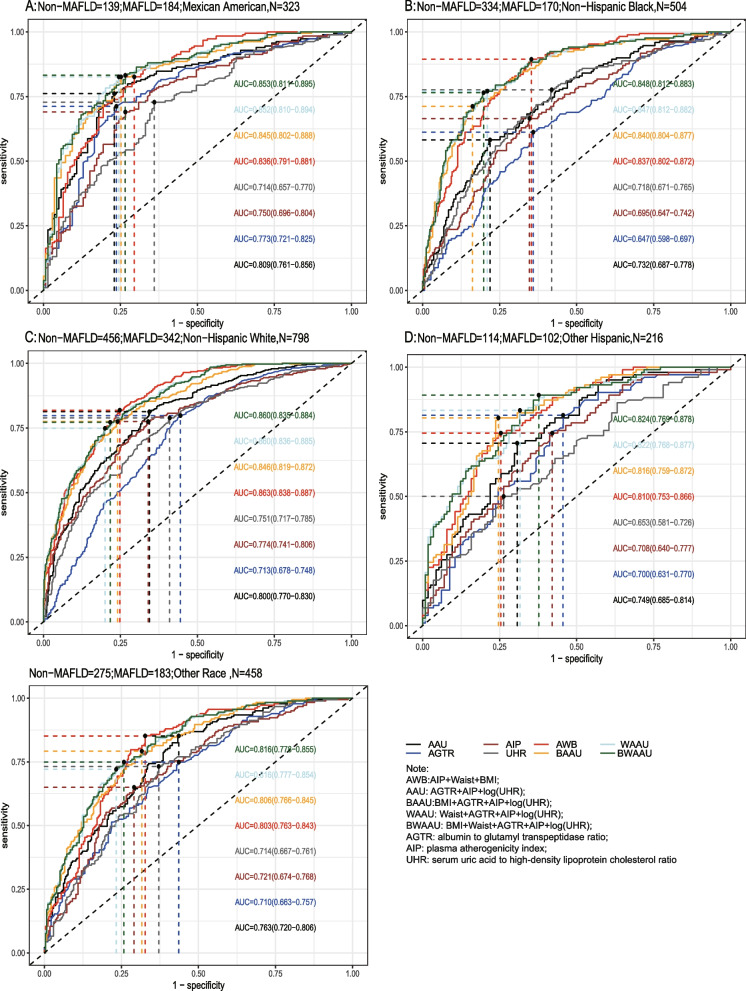
The second validation's roc curve for the subgroup

Using the test set extracted from five cycles of NHANES data from 2007–2016 for testing the predictive model, the receiver-operating characteristic curve (ROC) and detailed information are shown in Table 6 and Fig. 2A, demonstrating the predictive ability (AUC) of the AIP, AGTR, UHR, and the combination of all three for MAFLD in the following order: 0.749 (95% CI: 0.733–0.765), 0.773 (95% CI: 0.757–0.788), 0.728 (95% CI: 0.711–0.745), and 0.824 (95% CI: 0.810–0.837). The predictive ability of the first three alone for MAFLD was similar, whereas the combined model was stronger for MAFLD than for MAFLD alone, with the best cutoff value of the combined predictive model being 0.334 (sensitivity = 0.761, specificity = 0.739). To further validate the predictive ability of the model, the entire 2017–2018 cycle dataset was used for the secondary validation of the model, and the results are shown in Table 6 and Fig. 2B. In the secondary validation of the model, the ability of the three to jointly predict MAFLD (AUC = 0.775) was similar to that of the AIP (AUC = 0.743) individually, but they were all stronger than the predictive ability of the AGTR and UHR individually for MAFLD. To further distinguish the predictive ability of the above models for MAFLD, we also analyzed age(18 ≤ age < 65 and ≥ 65), BMI (< 25 kg/m2 and ≥ 25 kg/m2), sex(female and male), diabetic and race(Mexican American, Other Hispanic, Non-Hispanic White, Non-Hispanic Black and Other Race) populations in further subgroups, and the results (the internal validation and second validation's roc curve for the subgroup) are shown in (Figs. 3, 4 and 5) and (Figs 6, 7 and 8). In the subgroups, the combined prediction outperformed the three models independently, with the above model performing better for MAFLD prediction in female, nonoverweight and mexican american patients.

**Table 6:** The AUC data from train set, internal validation set and second validation set

	**AUC**	**Sensitivity**	**Specificity**	**Cutoff**	***p*-value**^**1**^
Train Set					
AAU	0.844	0.814	0.727	0.308	ref
AGTR	0.782	0.767	0.648	0.312	< 0.001
AIP	0.759	0.702	0.686	0.305	< 0.001
UHR	0.750	0.716	0.660	0.306	< 0.001
AWB	0.902	0.837	0.803	0.286	< 0.001
BAAU	0.915	0.882	0.786	0.271	< 0.001
WAAU	0.930	0.878	0.824	0.288	< 0.001
BWAAU	0.930	0.875	0.826	0.293	< 0.001
Internal validation					
AAU	0.824	0.761	0.739	0.334	ref
AGTR	0.773	0.680	0.721	0.368	< 0.001
AIP	0.749	0.687	0.707	0.317	< 0.001
UHR	0.728	0.684	0.651	0.309	< 0.001
AWB	0.894	0.841	0.772	0.268	< 0.001
BAAU	0.903	0.862	0.777	0.296	< 0.001
WAAU	0.920	0.843	0.840	0.359	< 0.001
BWAAU	0.920	0.852	0.831	0.343	< 0.001
Second validation					
AAU	0.775	0.761	0.656	0.301	ref
AGTR	0.697	0.792	0.507	0.263	< 0.001
AIP	0.743	0.733	0.643	0.301	< 0.001
UHR	0.722	0.704	0.638	0.287	< 0.001
AWB	0.831	0.792	0.726	0.247	< 0.001
BAAU	0.830	0.778	0.737	0.303	< 0.001
WAAU	0.837	0.812	0.710	0.222	< 0.001
BWAAU	0.837	0.804	0.717	0.236	< 0.001

*AWB* AIP + Waist + BMI, *AAU* AGTR + AIP + UHR, *BAAU *BMI + AGTR + AIP + log(UHR), *WAAU* Waist + AGTR + AIP + log(UHR), *BWAAU* BMI + Waist + AGTR + AIP + log(UHR), *AGTR* albumin to glutamyl transpeptidase ratio, *AIP* plasma atherogenicity index, *UHR* serum uric acid to high-density lipoprotein cholesterol ratio

^*^The logarithm is based on “e”

^1^Delong test

### Comparison of the combined model and available models for predicting MAFLD

In order to complement and improve the new model, we compared the established model with the existing model (A-W-B) [14] and added waist circumference and or body mass index (BMI) to the new model as a supplement. Delong Test was used to compare the models and the results of the analysis are shown in Table 6 and the parameter information of the model is shown in Table 7. We found that adding waist circumference and/or BMI to the joint prediction model improves the performance of the prediction, with excellent prediction performance in the internal test set, AUC > 0.90,and in the secondary validation set, AUC > 0.80. It is worth noting that the simultaneous inclusion of waist circumference and BMI in the model did not significantly improve the performance of the prediction model. Inclusion of waist circumference and/or BMI in the model was superior to the A-W-B model in the total population as well as in the subgroup analyses.

**Table 7:** Establishment of the logistic regression of all prediction models

Characteristic	OR^a^	95% CI^a^	*p*-value
AAU			
AGTR	0.36	0.33, 0.39	< 0.001
AIP	5.39	3.87, 7.50	< 0.001
log(UHR)	3.61	2.86, 4.56	< 0.001
AWB			
BMI	1.01	0.98, 1.03	0.6
AIP	12.5	9.20, 17.1	< 0.001
Waist	1.14	1.13, 1.16	< 0.001
BAAU			
BMI	1.29	1.27, 1.31	< 0.001
AGTR	0.33	0.29, 0.36	< 0.001
AIP	6.16	4.16, 9.14	< 0.001
log(UHR)	1.91	1.44, 2.54	< 0.001
WAAU			
Waist	1.15	1.14, 1.16	< 0.001
AGTR	0.33	0.29, 0.37	< 0.001
AIP	6.64	4.29, 10.3	< 0.001
log(UHR)	1.26	0.92, 1.74	0.2
BWAAU			
BMI	1.03	1.01, 1.06	0.020
Waist	1.13	1.12, 1.15	< 0.001
AGTR	0.33	0.29, 0.37	< 0.001
AIP	6.65	4.31, 10.3	< 0.001
log(UHR)	1.29	0.94, 1.78	0.12

*The logarithm is based on “e” *AWB *AIP+Waist+BMI, *AAU* AGTR+AIP+UHR, *BAAU* BMI+AGTR+AIP+log(UHR), *WAAU* Waist+AGTR+AIP+log(UHR), *BWAAU* BMI+Waist+AGTR+AIP+log(UHR), *AGTR* albumin to glutamyl transpeptidase ratio, *AIP,* plasma atherogenicity index, *UHR* serum uric acid to high-density lipoprotein cholesterol ratio

^a^*OR* = Odds Ratio, *CI* = Confidence Interval

## Discussion

The results of this study showed that a greater AGTR was beneficial in reducing the risk of developing MAFLD, with an OR of 0.31, indicating that for each unit increase, the risk of developing MAFLD was reduced by 69%, which is indicative of a strong independent protective factor, However, the results are based on cross-sectional studies, and multifactorial logistic regression analyses may be biased for non-rare diseases in cross-sectional studies [34]. Albumin is a biologically active substance synthesized by the liver and a marker of liver function with many biological functions. It is the most abundant plasma protein in human blood, transporting metals, fatty acids, cholesterol, bile pigments, and drugs, and it is also the main antioxidant in body fluids, playing an important anti-inflammatory role in inflammatory oxidative stress [35]. In the present study, albumin levels were lower in the MAFLD population than in the non-MAFLD population, which may be associated with the involvement of inflammation in the development of MAFLD, and lipid accumulation in the liver promotes the progression of hepatic inflammation [4], making the albumin level low in the MAFLD population. Albumin binds to free fatty acids and reduces the levels of free fatty acids, which are one of the important triggers of insulin resistance, and increased levels of free fatty acids can lead to deterioration of insulin sensitivity, while induction of tissue oxidative stress can lead to tissue insulin resistance [36]. Glutamyl transpeptidase (GGT) has good sensitivity for the diagnosis of NAFLD and is one of the indicators that make up the Fatty Liver Index (FLI), which is able to participate in the metabolic process of the glutathione antioxidant system; thus, GGT can be elevated in inflammatory states. It has been shown that GGT also increases the risk of insulin resistance, which is considered an important developmental factor in MAFLD [37]. Albumin has a negative correlation on GGT's. On the one hand, when albumin level decreases, free fatty acids are elevated, which will stimulate the synthesis and release of GGT [38]. On the other hand, the anti-inflammatory effect of albumin, which will inhibit the occurrence of oxidative stress, plays a protective role in the liver, thus reducing the risk of MAFLD. In addition, GGT is also an important indicator reflecting intrahepatic cholestasis, and the state of intrahepatic microcholestasis is involved in the development of MAFLD, so an elevated level of GGT or a decreased level of albumin will increase the risk of the development of MAFLD. We hypothesized that the AGTR would have predictive value for MAFLD. Our conjecture was revealed in both internal and secondary validation, showing that the AGTR was significantly better than the UHR in predicting MAFLD in the diabetic population, which indicated that it may be a potential inflammatory marker after the UHR and a more accessible and accurate predictive indicator for MAFLD patients. However, while previous studies have shown that 1/AGTR can be used as an independent predictor of coronary artery disease [39, 40], there are still few studies on the AGTR, and its predictive value in MAFLD or NAFLD has not yet been explored. Our study is the first to use the AGTR as a predictor of MAFLD and has emphasized the role of albumin in MAFLD, whose mechanism of action may be related to oxidative and antioxidant imbalance, but how albumin and GGT work together in MAFLD has not yet been clarified and needs to be further explored.

UHR is a relatively recent and novel marker of inflammation, consisting of uric acid as well as HDL, and it has been shown that high levels are associated with high abdominal visceral fat (VFA), which is associated with central obesity, a risk factor for the development of MAFLD [8, 41]. In addition, UHR may increase the burden of inflammation and oxidative stress, which indirectly affects the insulin sensitivity of patients, leading to the development of MAFLD. In the present study, the predictive value of UHR for MAFLD was explored, and multiple subgroup analyses showed that the female population had better predictive performance, which may be related to differences in hormone levels between genders. The results are consistent with previous studies [31].

AIP is a marker that responds to lipid metabolism, which is strongly associated with metabolic syndrome and the occurrence of adverse cardiovascular events; therefore, in this study, we evaluated the relationship between the AIP and MAFLD and demonstrated that the AIP was significantly and positively associated with the risk of developing MAFLD and could be used as a predictor of MAFLD. In the total population, our findings are compatible with a previous meta-analysis [12] showing the beneficial role of the AIP in predicting MAFLD or NAFLD with internal validation and secondary validation showing an AUC > 0.7. In subgroup analyses, the AIP predicted MAFLD better in nondiabetic than in diabetic populations. AIP not only increases the risk of insulin resistance [42], but also leads to disturbances in lipid metabolism. A retrospective study based on a Chinese diabetic population showed that the AIP has a predictive value in the diabetic population [13]. However, this study only evaluated the diabetic population and obtained a value of 0.57 for the resultant AUC, which is not a very good predictive performance. Our study is consistent with the findings of Duan, Shao-Jie et al. [14]. However, we added subgroup analyses of diabetic populations, ethnic populations, which allowed the predictive value of AIP to be validated in a wider population.

To further improve the prediction performance of the prediction model for MAFLD, we combined the AGTR, AIP, and UHR to jointly predict MAFLD, and through the complementary prediction performance of the three, the results showed that in the total population as well as subgroup analysis, the prediction of MAFLD by the combination of the three was stronger than that of the individual predictive ability, and our findings also showed that in the female population, the joint predictive ability of the three for MAFLD was the best among the subgroups. In the female population, the prevalence of NAFLD and MAFLD was lower than that in the male population, and estradiol had an antioxidant effect, whereas in the nonmenopausal female population, estradiol levels were higher than those in the male population, which may be attributed to the protective effect of estradiol [43]. Furthermore, estradiol reduces serum concentrations of GGT, uric acid and triglycerides and indirectly reduces diet-induced fatty liver injury via peroxidase [44–46]. These factors may explain why the AGTR, AIP, and UHR are better predictors in the female population.

Finally, we also compared the strengths and weaknesses of our model with the A-W-B model, and our study showed that our model supplemented with waist circumference and or BMI parameters was clearly superior to the A-W-B model in the total population as well as in each subgroup. Therefore, when using our model in the clinical setting, the addition of waist circumference or BMI can be a better predictor of MAFLD. Our study also showed that waist circumference is a better predictor of MAFLD than BMI, which may be that waist circumference is more reflective of central obesity [47], which is an important risk factor for MAFLD. Therefore, we recommend prioritizing the use of waist circumference over BMI when screening people for MAFLD [48]. The predictive model based on weighted analysis in this study has metrics that are easy to obtain, less costly than CT, MRI, and other imaging, easy to compute, and conducive to replication in physical exams or hospitalizations in the U.S. population.

Several advantages of this study are worth mentioning. To our knowledge, this is the first study to use the AGTR as a predictor of MAFLD. In addition, this is also the first study to assess the predictive efficacy of the AIP for MAFLD in the NHANES dataset. However, we acknowledge that there are also some limitations in this study, of which three main limitations were observed. First, this study is a cross-sectional study, which prevents us from drawing conclusions about causality. The longitudinal design will make the results more reliable. Second, the modalities we used to diagnose fatty liver were USFLI and transient elastography, and although their accuracy has been widely validated. We may underestimate the prevalence of MAFLD. Therefore, the gold standard is still liver puncture biopsy. Finally, some of the data used in the diagnosis of MAFLD were derived from a questionnaire, and the results may be somewhat biased. We may underestimate the impact of factors such as diet, exercise, and alcohol consumption on predictive markers. More prospective cohort studies are still needed to fully validate our findings.

## Conclusion

In conclusion, our study showed that the AGTR, AIP, and UHR have strong MAFLD predictive value and their combination can increase the predictive performance, especially in the female population. This study is important for developing personalized MAFLD diagnostic and treatment methods.

## Data Availability

The datasets generated and/or analysed during the current study are available in the NHANES repository, [https://wwwn.cdc.gov/nchs/nhanes/Default.aspx].

## Abbreviations

MAFLD: Metabolism dysfunction-associated fatty liver disease
NAFLD: Non-alcoholic liver disease
ALD: Alcoholic liver disease
NHANES: The National Health and Nutrition Examination Survey
NCHS: National Center for Health Statistics
CDC: Center for Disease Control and Prevention
CAP: Controlled attenuation parameter
ALT: Alanine Aminotransferase
AST: Aspartate Aminotransferase
BMI: Body Mass Index
HBA1C: Hemoglobin A1C
AGTR: Albumin to glutamyl transpeptidase ratio
AIP: Plasma atherogenicity index
UHR: Serum uric acid to high-density lipoprotein cholesterol ratio
ROC: Receiver Operating Characteristic Curve
HDL-C: High-density lipoprotein cholesterol
USFLI: United States Fatty Liver Index
